# Bis(2-{[3-methyl-4-(2,2,2-trifluoro­eth­oxy)-2-pyrid­yl]methyl­sulfan­yl}-1*H*,3*H* ^+^-benzimidazolium) 2,5-dichloro-3,6-dioxocyclo­hexa-1,4-diene-1,4-diolate

**DOI:** 10.1107/S1600536810019665

**Published:** 2010-05-29

**Authors:** Q. N. M. Hakim Al-arique, Jerry P. Jasinski, Ray J. Butcher, H. S. Yathirajan, B. Narayana

**Affiliations:** a Department of Studies in Chemistry, University of Mysore, Manasagangotri, Mysore 570 006, India; b Department of Chemistry, Keene State College, 229 Main Street, Keene, NH 03435-2001, USA; c Department of Chemistry, Howard University, 525 College Street NW, Washington, DC 20059, USA; d Department of Studies in Chemistry, Mangalore University, Mangalagangotri, 574 199, India

## Abstract

The title salt, 2C_16_H_15_F_3_N_3_OS^+^·C_6_Cl_2_O_4_^2−^, is composed of two independent cations of a lansoprazole {systematic name 2-([3-methyl-4-(2,2,2-trifluoroethoxy)pyridin-2-yl]methylsulfinyl)-1*H*-benzo[*d*]imidazole} inter­mediate and a dianion of chloranilic acid. In the cations of the lansoprazole inter­mediate, the dihedral angles between the least-squares planes of the pyridine and benzimidazole rings are 11.1 (6) and 13.1 (5)°, respectively. The dihedral angles between the mean plane of the benzene ring in the chloranilic acid dianion and the pryidine and benzimidazole rings of the two lansoprazole inter­mediate groups are 71.8 (1)/80.5 (7) and 74.2 (4)/74.8 (6)°. In addition to ionic bond inter­actions, the lansoprazole inter­mediate and chloranilic ions are connected by strong N—H⋯O hydrogen bonds, which produce a set of extended O—H⋯O—H⋯O—H chains along the *b* axis in the (011) plane. In addition, weak C—H⋯O, C—H⋯F, N—H⋯Cl and π–π [centroid–centroid distances = 3.5631 (15), 3.8187 (13), 3.7434 (17) and 3.842 (2) Å] inter­molecular inter­actions are observed, which contribute to crystal packing stability.

## Related literature

For bacterial growth inhibition by lansoprazole and its analogs, see: Iwahi *et al.* (1991 ▶). For related structures, see: Arslan *et al.* (2006 ▶); Gotoh *et al.* (2006 ▶, 2007 ▶, 2008 ▶); Ishida (2004*a* ▶,*b* ▶,*c* ▶); Ishida & Kashino (1999 ▶, 2000 ▶); Meng & Qian (2006 ▶); Refat *et al.* (2006 ▶); Swamy & Ravikumar (2007 ▶); Tabuchi *et al.* (2005 ▶); Vyas *et al.* (2000 ▶).
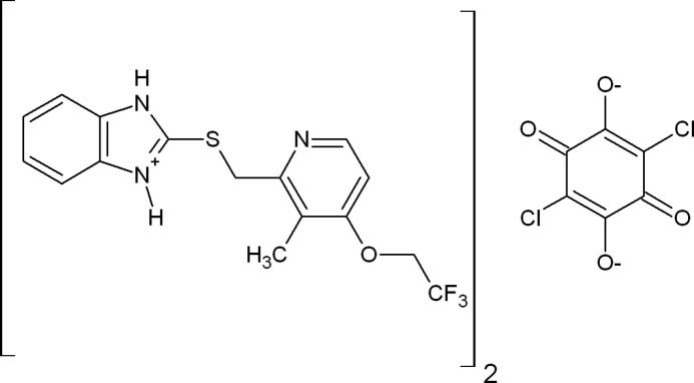


## Experimental

### 

#### Crystal data

2C_16_H_15_F_3_N_3_OS^+^·C_6_Cl_2_O_4_^2−^
*M* *_r_* = 915.70
Monoclinic, 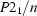

*a* = 9.48575 (8) Å
*b* = 23.6316 (2) Å
*c* = 17.86775 (15) Å
β = 100.2065 (9)°
*V* = 3941.92 (6) Å^3^
*Z* = 4
Cu *K*α radiation
μ = 3.22 mm^−1^
*T* = 295 K
0.38 × 0.24 × 0.19 mm

#### Data collection

Oxford Diffraction Xcalibur Ruby Gemini diffractometer
Absorption correction: multi-scan (*CrysAlis RED*; Oxford Diffraction, 2007 ▶) *T*_min_ = 0.692, *T*_max_ = 1.000
19572 measured reflections
8269 independent reflections
6572 reflections with *I* > 2σ(*I*)
*R*_int_ = 0.019

#### Refinement

*R*[*F* ^2^ > 2σ(*F* ^2^)] = 0.050
*wR*(*F* ^2^) = 0.158
*S* = 1.10
8269 reflections
600 parameters
138 restraints
H-atom parameters constrained
Δρ_max_ = 0.87 e Å^−3^
Δρ_min_ = −0.49 e Å^−3^

### 

Data collection: *CrysAlis PRO* (Oxford Diffraction, 2007 ▶); cell refinement: *CrysAlis PRO*; data reduction: *CrysAlis PRO*; program(s) used to solve structure: *SHELXS97* (Sheldrick, 2008 ▶); program(s) used to refine structure: *SHELXL97* (Sheldrick, 2008 ▶); molecular graphics: *SHELXTL* (Sheldrick, 2008 ▶); software used to prepare material for publication: *SHELXTL*.

## Supplementary Material

Crystal structure: contains datablocks global, I. DOI: 10.1107/S1600536810019665/bt5269sup1.cif

Structure factors: contains datablocks I. DOI: 10.1107/S1600536810019665/bt5269Isup2.hkl

Additional supplementary materials:  crystallographic information; 3D view; checkCIF report

## Figures and Tables

**Table 1:** Hydrogen-bond geometry (Å, °)

*D*—H⋯*A*	*D*—H	H⋯*A*	*D*⋯*A*	*D*—H⋯*A*
N1*A*—H1*AA*⋯O3	0.86	1.95	2.749 (2)	155
N1*A*—H1*AA*⋯Cl2	0.86	2.96	3.5169 (18)	125
N2*A*—H2*AA*⋯O5^i^	0.86	1.91	2.737 (2)	160
N1*B*—H1*BA*⋯O2	0.86	1.89	2.717 (2)	160
N2*B*—H2*BA*⋯O6^i^	0.86	1.96	2.766 (2)	155
C8*A*—H8*AB*⋯O5^i^	0.97	2.50	3.195 (3)	127
C8*B*—H8*BA*⋯O6^i^	0.97	2.45	3.289 (3)	145
C6*B*—H6*BA*⋯F3*AA*^ii^	0.93	2.49	3.104 (5)	124

Symmetry codes: (i) 
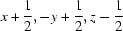
; (ii) 
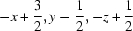
.

**Fig. 1.**
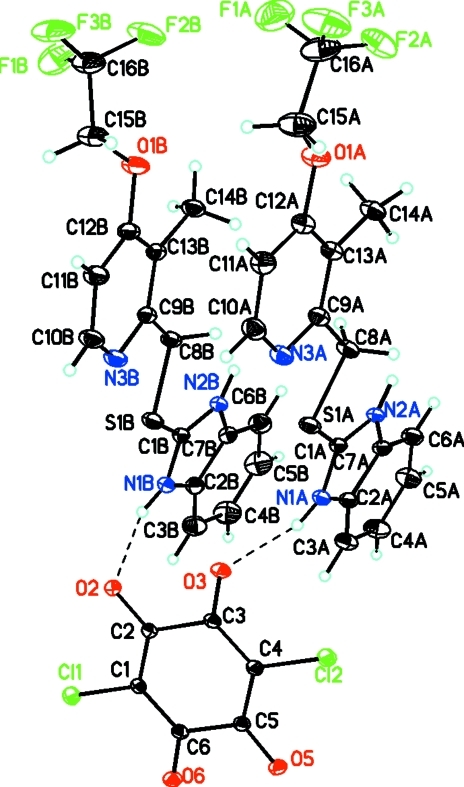
Molecular structure of [C16H15F3N3OS+]2 [C6Cl2O42-], showing the atom labeling scheme and 50% probability displacement ellipsoids. Dashed lines indicate strong N—H···O intermolecular interactions within the asymmetric unit. H atoms are presented as small circles of arbitrary radius.

**Fig. 2.**
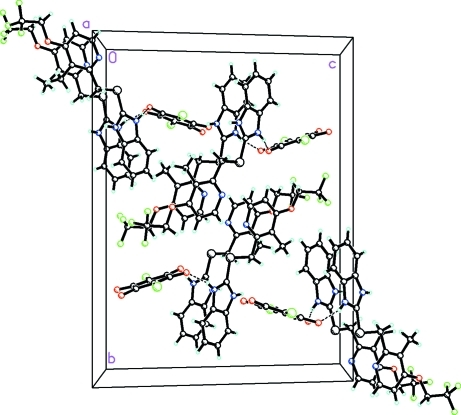
Packing diagram of the title compound ,[C16H15F3N3OS+]2 [C6Cl2O42-],, viewed down the a axis. Dashed lines indicate strong N—H···O and weak C—H···O, C—H···F intermolecular hydrogen bonds linking the [C16H15F3N3OS+]2 and [C6Cl2O42-] ions into an infinite O—H···O—H···O—H chain network along the b axis in the (011) plane.

## supplementary crystallographic information

### Comment

The Lansoprazole intermediate (Systematic name: 2-([[3-Methyl-4-(2,2,2-trifluoroethoxy)-2-pyridinyl]methyl]sulfanyl) -1*H*-benzimidazole) in this study is a benzimidazole derivative. Lansoprazole, a widely used proton-pump inhibitor, has been reported to have an independent gastroprotective action. Lansoprazole and its analogs inhibit the growth of *Helicobacter pylori* at concentrations of several micrograms per milliliter (Iwahi *et al.*, 1991) and is widely used for the treatment of acid-related gastric diseases due to their ability to inhibit acid secretio.   The crystal structures of lansoprazole (Vyas *et al.*, 2000) and lansoprazole sulphone have been reported (Swamy & Ravikumar, 2007).

Charge transfer complexes of organic species are intensively studied because of their special type of interaction, which is accompanied by transfer of an electron from the donor to the acceptor. Chloranilic acid is a strong dibasic organic acid which exhibits the electron-acceptor properties on one hand and acidic properties leading to formation of hydrogen bonds on the other hand. In the case of stronger bases the proton-transfer hydrogen bonded ion pairs will be formed which is interesting from the point of view of electron transfer reactions in biological systems. Protonation of the donor from acidic acceptors are generally a route for the formation of ion pair adducts. The synthesis and spectroscopic studies of charge transfer complexes between chloranilic acid and some heterocyclic amines in ethanol and amino heterocyclic donors in acteonitrile have been studied. The interaction of the lansoprazole intermediate as an electron donor with chloranilic acid as electron acceptor in this study resulted in the formation of a charge transfer complex of the title compound (I). In view of the importance of lansoprazole, this paper reports the crystal structure of [C_16_H_15_F_3_N_3_OS+]_2_ [C_6_Cl_2_O_4_^2-^], (I).

The title compound (I) is a salt composed of two independent cations (A & B) of a lansoprazole intermediate, [C_16_H_15_F_3_N_3_OS+]_2_, and a dianion [C_6_Cl_2_O_4_^2-^] of chloranilic acid, (2:1) in the asymmetric unit (Fig. 1). In each cation (A & B) of the lansoprazole intermediate the dihedral angles between the least squares planes of the pyridine and benzimidazole rings are 11.1 (6)° (A) and 13.1 (5)° (B), respectively. The dihedral angles between the mean plane of the benzene ring in the chloranilic acid dianion and the pryidine and benzimidazole rings of the two lansoprazole intermediate groups are 71.8 (1)° (A), 80.5 (7)° (B) and 74.2 (4)° (A), 74.8 (6)° (B), respectively. The fluorine atoms in both cations are disordered (relative occupancies = 0.361 (5) (A), 0.639 (5) (A) and 0.684 (5) (B), 0.316 (5) (B)). In neutral chloranilic acid, typical C═O and C–O(—H) bond lengths are 1.22 (1)Å and 1.32 (1) Å. For the chloroanilate monoanion C═O and C–O^-^, values of 1.24 (2)Å and 1.25 (2) Å have been reported (Gotoh *et al.*, (2007). In (I), we report values of 1.248 (2)Å (C2═O2), 1.248 (2) Å (C5═O5), 1.249 (2) Å (C3–O3^-^) and 1.245 (2) Å (C6–O6^-^), respectively. In addition to ionic bond interactions, the lansoprazole intermediate and chloranilic acid ions are connected by strong N—H···O hydrogen bonds [N1A···O3 = 2.749 (2) Å; N2A···O5 = 2.737 (2) Å; N1B···O2 = 2.717 (2) Å; N2B···O6 = 2.766 (2) Å] (Fig. 2, Table 1). This produces a set of O—H···O—H···O—H infinite one-dimensional chains along the *b* axis in the (011) plane. In addition, weak C—H···O, C—H···F, N—H···Cl (Table 1) and π–π (Table 2) intermolecular stacking interactions are observed which contribute to crystal packing stability (Fig. 2).

### Experimental

The title compound was synthesized by adding a saturated solution of chloranilic acid (0.42 g, 2 mmol) in methanol (10 ml) to a solution of the lansoprazole intermediate (0.74 g, 2 mmol) in methanol (10 ml). A red color developed and the solution was allowed to evaporate slowly at room temperature. The red color complex formed was filtered off, washed with diethyl ether and dried under vacuum (Yield: 72.4%). Crystals were grown from a dimethyl formamide solution (m.p.: 441-444 K). Composition (%) found (calculated) for [C_16_H_15_F_3_N_3_OS+]_2_ [C_6_Cl_2_O_4_^2-^]: C: 49.76 (49.84); H: 3.28 (3.30); N: 9.15 (9.18).

### Refinement

The H atoms were placed in geometrically idealized positions and constrained to ride on their parent atoms with N–H = 0.86Å and C–H distances in the range 0.93-0.97Å and with U_iso_(H) = 1.19-1.50 U_eq_(C,N). The fluorine atoms were disordered with F1A, F2A, F3A at 0.361 (5) and F1AA, F2AA, F3AA at 0.639 (5) partial occupancy. F1B, F2B, F3B were placed at 0.684 (5) and F1BB, F2BB, F3BB at 0.316 (5) partial occupancy. All fluorine atoms were then refined anisotropically. The following restraints were applied: the ellipsoids of the F atoms were restrained to be isotropic and to have a similar shape than their opposite counterpart. The C-F distances were restrained to 1.303 (1)Å and the F···F distances to 2.128 (1)Å.

### Crystal data

**Table:**

2C_16_H_15_F_3_N_3_OS^+^·C_6_Cl_2_O_4_^2^^−^	*F*(000) = 1872
*M**_r_* = 915.70	*D*_x_ = 1.543 Mg m^−^^3^
Monoclinic, *P*2_1_/*n*	Cu *K*α radiation, λ = 1.54184 Å
Hall symbol: -P 2yn	Cell parameters from 10683 reflections
*a* = 9.48575 (8) Å	θ = 4.5–77.4°
*b* = 23.6316 (2) Å	µ = 3.22 mm^−^^1^
*c* = 17.86775 (15) Å	*T* = 295 K
β = 100.2065 (9)°	Prism, red-brown
*V* = 3941.92 (6) Å^3^	0.38 × 0.24 × 0.19 mm
*Z* = 4	

### Data collection

**Table:**

Oxford Diffraction Xcalibur Ruby Gemini diffractometer	8269 independent reflections
Radiation source: Enhance (Cu) X-ray Source	6572 reflections with *I* > 2σ(*I*)
graphite	*R*_int_ = 0.019
Detector resolution: 10.5081 pixels mm^-1^	θ_max_ = 77.6°, θ_min_ = 4.5°
ω scans	*h* = −11→10
Absorption correction: multi-scan (*CrysAlis RED*; Oxford Diffraction, 2007)	*k* = −29→21
*T*_min_ = 0.692, *T*_max_ = 1.000	*l* = −21→22
19572 measured reflections	

### Refinement

**Table:**

Refinement on *F*^2^	Primary atom site location: structure-invariant direct methods
Least-squares matrix: full	Secondary atom site location: difference Fourier map
*R*[*F*^2^ > 2σ(*F*^2^)] = 0.050	Hydrogen site location: inferred from neighbouring sites
*wR*(*F*^2^) = 0.158	H-atom parameters constrained
*S* = 1.10	*w* = 1/[σ^2^(*F*_o_^2^) + (0.1054*P*)^2^ + 0.2084*P*] where *P* = (*F*_o_^2^ + 2*F*_c_^2^)/3
8269 reflections	(Δ/σ)_max_ = 0.007
600 parameters	Δρ_max_ = 0.87 e Å^−^^3^
138 restraints	Δρ_min_ = −0.49 e Å^−^^3^

### Special details

**Table:**

Geometry. All esds (except the esd in the dihedral angle between two l.s. planes) are estimated using the full covariance matrix. The cell esds are taken into account individually in the estimation of esds in distances, angles and torsion angles; correlations between esds in cell parameters are only used when they are defined by crystal symmetry. An approximate (isotropic) treatment of cell esds is used for estimating esds involving l.s. planes.
Refinement. Refinement of *F*^2^ against ALL reflections. The weighted *R*-factor *wR* and goodness of fit *S* are based on *F*^2^, conventional *R*-factors *R* are based on *F*, with *F* set to zero for negative *F*^2^. The threshold expression of *F*^2^ > σ(*F*^2^) is used only for calculating *R*-factors(gt) *etc*. and is not relevant to the choice of reflections for refinement. *R*-factors based on *F*^2^ are statistically about twice as large as those based on *F*, and *R*- factors based on ALL data will be even larger.

### Fractional atomic coordinates and isotropic or equivalent isotropic displacement parameters (Å^2^)

**Table:**

	*x*	*y*	*z*	*U*_iso_*/*U*_eq_	Occ. (<1)
Cl1	−0.02389 (6)	0.28125 (3)	0.75775 (3)	0.06238 (17)	
Cl2	0.65326 (6)	0.29242 (4)	0.82221 (3)	0.06540 (18)	
O2	0.16831 (17)	0.31643 (8)	0.65093 (9)	0.0562 (4)	
O3	0.45465 (16)	0.32221 (7)	0.67875 (8)	0.0499 (4)	
O5	0.45948 (16)	0.26157 (7)	0.92935 (8)	0.0487 (3)	
O6	0.17567 (16)	0.25255 (8)	0.90012 (9)	0.0518 (4)	
C1	0.1616 (2)	0.28648 (9)	0.77553 (11)	0.0401 (4)	
C2	0.2306 (2)	0.30329 (8)	0.71610 (11)	0.0387 (4)	
C3	0.3966 (2)	0.30515 (8)	0.73206 (11)	0.0380 (4)	
C4	0.4674 (2)	0.28877 (9)	0.80369 (11)	0.0410 (4)	
C5	0.3979 (2)	0.27343 (8)	0.86360 (10)	0.0372 (4)	
C6	0.2333 (2)	0.27039 (8)	0.84730 (11)	0.0368 (4)	
S1A	0.76555 (7)	0.36213 (3)	0.55737 (3)	0.05601 (14)	
F1A	0.9879 (2)	0.53715 (13)	0.14545 (16)	0.161 (4)	0.361 (5)
F2A	1.1807 (2)	0.50633 (8)	0.2122 (2)	0.105 (2)	0.361 (5)
F3A	1.1443 (3)	0.59488 (7)	0.2010 (3)	0.149 (3)	0.361 (5)
F1AA	1.0095 (2)	0.51401 (8)	0.15374 (14)	0.1149 (16)	0.639 (5)
F2AA	1.21213 (17)	0.52535 (11)	0.2249 (2)	0.153 (2)	0.639 (5)
F3AA	1.0897 (4)	0.59702 (6)	0.1800 (2)	0.216 (3)	0.639 (5)
O1A	0.9921 (3)	0.49361 (8)	0.29873 (12)	0.0721 (5)	
N1A	0.68513 (19)	0.27244 (9)	0.63087 (10)	0.0479 (4)	
H1AA	0.6338	0.2931	0.6552	0.057*	
N2A	0.8291 (2)	0.24764 (8)	0.55373 (10)	0.0469 (4)	
H2AA	0.8853	0.2497	0.5210	0.056*	
N3A	0.8120 (2)	0.45798 (9)	0.48750 (13)	0.0592 (5)	
C1A	0.7631 (2)	0.29124 (10)	0.58035 (11)	0.0450 (5)	
C2A	0.7007 (2)	0.21455 (11)	0.63736 (12)	0.0496 (5)	
C3A	0.6439 (3)	0.17509 (13)	0.68139 (16)	0.0650 (7)	
H3AA	0.5843	0.1858	0.7150	0.078*	
C4A	0.6802 (4)	0.11919 (14)	0.67281 (19)	0.0813 (9)	
H4AA	0.6447	0.0916	0.7016	0.098*	
C5A	0.7691 (4)	0.10301 (14)	0.62188 (19)	0.0781 (8)	
H5AA	0.7894	0.0648	0.6169	0.094*	
C6A	0.8277 (3)	0.14224 (12)	0.57871 (15)	0.0634 (6)	
H6AA	0.8879	0.1315	0.5454	0.076*	
C7A	0.7919 (2)	0.19858 (11)	0.58771 (12)	0.0492 (5)	
C8A	0.8823 (3)	0.36051 (11)	0.48755 (15)	0.0570 (6)	
H8AA	0.9794	0.3513	0.5116	0.068*	
H8AB	0.8497	0.3323	0.4490	0.068*	
C9A	0.8769 (3)	0.41856 (10)	0.45245 (14)	0.0517 (5)	
C10A	0.8074 (3)	0.51054 (12)	0.45988 (16)	0.0653 (7)	
H10A	0.7620	0.5382	0.4840	0.078*	
C11A	0.8657 (3)	0.52621 (12)	0.39785 (16)	0.0641 (6)	
H11A	0.8612	0.5634	0.3807	0.077*	
C12A	0.9315 (3)	0.48433 (11)	0.36190 (15)	0.0577 (6)	
C13A	0.9393 (3)	0.42859 (10)	0.38825 (14)	0.0526 (5)	
C14A	1.0114 (3)	0.38352 (12)	0.34966 (17)	0.0673 (7)	
H14A	1.0194	0.3496	0.3797	0.101*	
H14B	1.1052	0.3961	0.3442	0.101*	
H14C	0.9558	0.3759	0.3004	0.101*	
C15A	1.0090 (6)	0.54929 (14)	0.2751 (2)	0.0985 (12)	
H15A	1.0667	0.5708	0.3156	0.118*	
H15B	0.9165	0.5676	0.2615	0.118*	
C16A	1.08337 (16)	0.54588 (6)	0.20623 (10)	0.1085 (15)	
S1B	0.37775 (7)	0.35551 (3)	0.47949 (3)	0.05589 (14)	
F1B	0.46185 (16)	0.52009 (8)	0.0783 (2)	0.144 (2)	0.684 (5)
F2B	0.68815 (15)	0.52999 (10)	0.10037 (18)	0.1317 (14)	0.684 (5)
F3B	0.5535 (3)	0.60246 (6)	0.08372 (17)	0.1343 (16)	0.684 (5)
F1BB	0.5025 (3)	0.59349 (8)	0.0729 (2)	0.114 (3)	0.316 (5)
F2BB	0.5274 (3)	0.50406 (7)	0.0777 (2)	0.115 (3)	0.316 (5)
F3BB	0.70535 (15)	0.55741 (14)	0.1178 (3)	0.162 (4)	0.316 (5)
O1B	0.5855 (3)	0.50090 (9)	0.22645 (11)	0.0748 (6)	
N1B	0.2888 (2)	0.26340 (9)	0.54297 (11)	0.0492 (4)	
H1BA	0.2374	0.2835	0.5679	0.059*	
N2B	0.4330 (2)	0.24045 (8)	0.46559 (10)	0.0467 (4)	
H2BA	0.4892	0.2434	0.4329	0.056*	
N3B	0.4174 (3)	0.45403 (9)	0.41534 (12)	0.0618 (5)	
C1B	0.3674 (2)	0.28334 (10)	0.49428 (11)	0.0446 (4)	
C2B	0.3029 (3)	0.20543 (11)	0.54709 (14)	0.0540 (5)	
C3B	0.2441 (4)	0.16568 (14)	0.58983 (19)	0.0760 (8)	
H3BA	0.1832	0.1759	0.6230	0.091*	
C4B	0.2813 (5)	0.10991 (17)	0.5801 (2)	0.0975 (12)	
H4BA	0.2449	0.0817	0.6077	0.117*	
C5B	0.3720 (5)	0.09494 (15)	0.5299 (3)	0.0991 (13)	
H5BA	0.3935	0.0569	0.5245	0.119*	
C6B	0.4311 (4)	0.13501 (13)	0.48792 (18)	0.0744 (8)	
H6BA	0.4922	0.1247	0.4549	0.089*	
C7B	0.3950 (3)	0.19064 (11)	0.49732 (13)	0.0526 (5)	
C8B	0.4554 (3)	0.35551 (11)	0.39369 (13)	0.0556 (6)	
H8BA	0.5487	0.3375	0.4032	0.067*	
H8BB	0.3941	0.3350	0.3535	0.067*	
C9B	0.4691 (3)	0.41622 (10)	0.37107 (12)	0.0495 (5)	
C10B	0.4281 (4)	0.50877 (12)	0.39791 (16)	0.0680 (7)	
H10B	0.3962	0.5355	0.4293	0.082*	
C11B	0.4831 (3)	0.52773 (12)	0.33653 (15)	0.0652 (7)	
H11B	0.4876	0.5662	0.3260	0.078*	
C12B	0.5319 (3)	0.48743 (11)	0.29059 (13)	0.0566 (6)	
C13B	0.5295 (3)	0.43033 (10)	0.30822 (12)	0.0513 (5)	
C14B	0.5910 (3)	0.38603 (12)	0.26233 (15)	0.0655 (7)	
H14D	0.6283	0.4040	0.2218	0.098*	
H14E	0.5171	0.3598	0.2415	0.098*	
H14F	0.6667	0.3661	0.2946	0.098*	
C15B	0.5554 (4)	0.55470 (13)	0.19478 (18)	0.0803 (9)	
H15C	0.6229	0.5822	0.2207	0.096*	
H15D	0.4595	0.5663	0.2001	0.096*	
C16B	0.56725 (15)	0.55150 (6)	0.11217 (10)	0.0924 (11)	

### Atomic displacement parameters (Å^2^)

**Table:**

	*U*^11^	*U*^22^	*U*^33^	*U*^12^	*U*^13^	*U*^23^
Cl1	0.0340 (2)	0.1034 (5)	0.0508 (3)	−0.0013 (2)	0.0106 (2)	0.0039 (3)
Cl2	0.0347 (2)	0.1177 (5)	0.0455 (3)	−0.0089 (3)	0.0117 (2)	0.0020 (3)
O2	0.0479 (8)	0.0864 (11)	0.0365 (7)	0.0193 (8)	0.0130 (6)	0.0129 (7)
O3	0.0493 (7)	0.0665 (9)	0.0389 (7)	−0.0025 (7)	0.0212 (6)	0.0073 (6)
O5	0.0436 (7)	0.0706 (9)	0.0325 (7)	−0.0026 (7)	0.0081 (6)	0.0078 (6)
O6	0.0443 (7)	0.0765 (10)	0.0379 (7)	−0.0111 (7)	0.0164 (6)	0.0072 (7)
C1	0.0313 (8)	0.0535 (10)	0.0374 (9)	−0.0002 (7)	0.0110 (7)	0.0003 (8)
C2	0.0398 (9)	0.0452 (9)	0.0334 (9)	0.0063 (7)	0.0127 (7)	0.0008 (7)
C3	0.0396 (9)	0.0429 (9)	0.0346 (9)	−0.0001 (7)	0.0147 (7)	0.0005 (7)
C4	0.0321 (8)	0.0577 (11)	0.0354 (9)	−0.0044 (8)	0.0117 (7)	0.0010 (8)
C5	0.0382 (9)	0.0430 (9)	0.0320 (8)	−0.0026 (7)	0.0106 (7)	0.0008 (7)
C6	0.0378 (9)	0.0408 (9)	0.0341 (8)	−0.0045 (7)	0.0130 (7)	−0.0013 (7)
S1A	0.0645 (3)	0.0645 (3)	0.0451 (3)	0.0095 (3)	0.0264 (2)	−0.0001 (2)
F1A	0.162 (7)	0.194 (7)	0.125 (6)	−0.033 (6)	0.018 (5)	0.057 (6)
F2A	0.113 (4)	0.084 (3)	0.136 (5)	−0.031 (3)	0.073 (4)	−0.031 (3)
F3A	0.227 (6)	0.088 (4)	0.166 (6)	−0.049 (4)	0.124 (5)	0.007 (4)
F1AA	0.176 (4)	0.110 (3)	0.0567 (18)	0.033 (3)	0.016 (2)	−0.0122 (17)
F2AA	0.109 (3)	0.207 (5)	0.149 (4)	−0.066 (3)	0.042 (3)	−0.009 (4)
F3AA	0.442 (8)	0.104 (4)	0.136 (4)	−0.017 (5)	0.142 (5)	0.011 (3)
O1A	0.0979 (14)	0.0607 (10)	0.0668 (11)	−0.0055 (10)	0.0391 (10)	0.0041 (9)
N1A	0.0432 (9)	0.0682 (11)	0.0350 (8)	−0.0004 (8)	0.0144 (7)	−0.0060 (8)
N2A	0.0453 (8)	0.0673 (11)	0.0309 (8)	0.0023 (8)	0.0142 (7)	−0.0026 (7)
N3A	0.0671 (12)	0.0609 (11)	0.0552 (11)	0.0049 (10)	0.0261 (9)	−0.0069 (9)
C1A	0.0412 (9)	0.0653 (12)	0.0298 (8)	0.0003 (9)	0.0097 (7)	−0.0036 (8)
C2A	0.0472 (11)	0.0671 (13)	0.0361 (10)	−0.0062 (9)	0.0119 (8)	−0.0051 (9)
C3A	0.0706 (15)	0.0759 (16)	0.0553 (13)	−0.0119 (13)	0.0301 (12)	−0.0056 (12)
C4A	0.107 (2)	0.0754 (18)	0.0710 (18)	−0.0169 (17)	0.0404 (17)	−0.0002 (15)
C5A	0.101 (2)	0.0652 (16)	0.0752 (18)	−0.0023 (15)	0.0363 (17)	−0.0048 (14)
C6A	0.0743 (16)	0.0688 (15)	0.0511 (13)	0.0052 (12)	0.0219 (12)	−0.0066 (11)
C7A	0.0501 (11)	0.0659 (13)	0.0330 (9)	−0.0028 (10)	0.0108 (8)	−0.0056 (9)
C8A	0.0640 (13)	0.0591 (13)	0.0554 (13)	0.0039 (11)	0.0313 (11)	−0.0038 (10)
C9A	0.0541 (11)	0.0573 (12)	0.0470 (11)	0.0001 (10)	0.0180 (9)	−0.0068 (9)
C10A	0.0741 (16)	0.0620 (14)	0.0636 (15)	0.0097 (12)	0.0230 (13)	−0.0104 (12)
C11A	0.0773 (16)	0.0535 (13)	0.0636 (15)	0.0023 (12)	0.0186 (13)	−0.0012 (11)
C12A	0.0625 (13)	0.0615 (13)	0.0522 (12)	−0.0056 (11)	0.0192 (10)	−0.0022 (10)
C13A	0.0557 (12)	0.0576 (12)	0.0488 (11)	−0.0010 (10)	0.0208 (10)	−0.0052 (10)
C14A	0.0813 (16)	0.0654 (15)	0.0651 (15)	0.0067 (13)	0.0399 (13)	−0.0019 (12)
C15A	0.162 (4)	0.0620 (17)	0.083 (2)	−0.012 (2)	0.054 (2)	0.0002 (16)
C16A	0.184 (5)	0.072 (2)	0.076 (2)	−0.025 (3)	0.040 (3)	0.0107 (18)
S1B	0.0684 (3)	0.0580 (3)	0.0482 (3)	0.0082 (3)	0.0293 (2)	0.0006 (2)
F1B	0.211 (5)	0.118 (3)	0.091 (3)	−0.007 (3)	−0.006 (3)	−0.012 (2)
F2B	0.191 (3)	0.118 (3)	0.117 (2)	0.027 (3)	0.110 (2)	0.008 (2)
F3B	0.265 (5)	0.0703 (18)	0.091 (2)	0.014 (2)	0.094 (3)	0.0221 (16)
F1BB	0.135 (6)	0.123 (6)	0.083 (5)	0.006 (5)	0.017 (4)	0.047 (4)
F2BB	0.188 (7)	0.108 (5)	0.053 (3)	0.038 (5)	0.028 (4)	−0.009 (3)
F3BB	0.150 (7)	0.205 (9)	0.149 (7)	−0.009 (6)	0.076 (6)	0.030 (7)
O1B	0.1124 (15)	0.0664 (11)	0.0570 (10)	0.0159 (11)	0.0467 (10)	0.0109 (8)
N1B	0.0446 (9)	0.0646 (11)	0.0415 (9)	0.0019 (8)	0.0158 (7)	−0.0003 (8)
N2B	0.0459 (9)	0.0610 (10)	0.0353 (8)	0.0045 (8)	0.0124 (7)	−0.0030 (7)
N3B	0.0853 (14)	0.0589 (11)	0.0494 (10)	0.0164 (10)	0.0340 (10)	0.0038 (9)
C1B	0.0416 (9)	0.0599 (12)	0.0332 (9)	0.0020 (8)	0.0088 (7)	−0.0019 (8)
C2B	0.0521 (12)	0.0662 (14)	0.0443 (11)	−0.0036 (10)	0.0103 (9)	−0.0003 (10)
C3B	0.0829 (18)	0.0791 (18)	0.0714 (18)	−0.0151 (15)	0.0283 (15)	0.0081 (15)
C4B	0.123 (3)	0.079 (2)	0.096 (3)	−0.018 (2)	0.036 (2)	0.0131 (19)
C5B	0.143 (4)	0.0581 (17)	0.097 (3)	0.000 (2)	0.024 (3)	0.0009 (17)
C6B	0.094 (2)	0.0654 (16)	0.0653 (17)	0.0083 (15)	0.0192 (15)	−0.0082 (13)
C7B	0.0550 (12)	0.0615 (13)	0.0408 (11)	0.0016 (10)	0.0071 (9)	−0.0021 (9)
C8B	0.0700 (14)	0.0589 (13)	0.0435 (11)	0.0150 (11)	0.0257 (10)	0.0054 (9)
C9B	0.0563 (11)	0.0569 (12)	0.0378 (10)	0.0129 (10)	0.0151 (9)	0.0019 (9)
C10B	0.0955 (18)	0.0612 (14)	0.0562 (13)	0.0193 (13)	0.0377 (13)	−0.0011 (11)
C11B	0.0927 (19)	0.0547 (13)	0.0548 (13)	0.0128 (13)	0.0310 (13)	0.0052 (11)
C12B	0.0704 (14)	0.0635 (13)	0.0404 (11)	0.0083 (11)	0.0223 (10)	0.0047 (10)
C13B	0.0613 (12)	0.0585 (12)	0.0363 (10)	0.0128 (10)	0.0148 (9)	0.0012 (9)
C14B	0.0878 (17)	0.0666 (15)	0.0495 (12)	0.0172 (13)	0.0321 (12)	−0.0007 (11)
C15B	0.129 (3)	0.0583 (15)	0.0645 (16)	−0.0002 (16)	0.0478 (17)	0.0028 (12)
C16B	0.148 (3)	0.0671 (18)	0.075 (2)	0.002 (2)	0.054 (2)	0.0131 (16)

### Geometric parameters (Å, °)

**Table:**

Cl1—C1	1.7363 (19)	C14A—H14A	0.9600
Cl2—C4	1.7370 (19)	C14A—H14B	0.9600
O2—C2	1.248 (2)	C14A—H14C	0.9600
O3—C3	1.249 (2)	C15A—C16A	1.525 (4)
O5—C5	1.248 (2)	C15A—H15A	0.9700
O6—C6	1.245 (2)	C15A—H15B	0.9700
C1—C6	1.394 (3)	S1B—C1B	1.731 (2)
C1—C2	1.400 (3)	S1B—C8B	1.814 (2)
C2—C3	1.551 (3)	F1B—C16B	1.3048 (11)
C3—C4	1.391 (3)	F2B—C16B	1.3053 (11)
C4—C5	1.401 (3)	F3B—C16B	1.3047 (11)
C5—C6	1.539 (3)	F1BB—C16B	1.3039 (12)
S1A—C1A	1.726 (2)	F2BB—C16B	1.3026 (12)
S1A—C8A	1.809 (2)	F3BB—C16B	1.3034 (11)
F1A—C16A	1.3016 (12)	O1B—C12B	1.371 (3)
F2A—C16A	1.3045 (11)	O1B—C15B	1.400 (4)
F3A—C16A	1.3052 (12)	N1B—C1B	1.328 (3)
F1AA—C16A	1.3056 (11)	N1B—C2B	1.377 (3)
F2AA—C16A	1.3016 (11)	N1B—H1BA	0.8600
F3AA—C16A	1.3014 (12)	N2B—C1B	1.338 (3)
O1A—C12A	1.372 (3)	N2B—C7B	1.382 (3)
O1A—C15A	1.400 (4)	N2B—H2BA	0.8600
N1A—C1A	1.340 (3)	N3B—C10B	1.339 (4)
N1A—C2A	1.379 (3)	N3B—C9B	1.342 (3)
N1A—H1AA	0.8600	C2B—C3B	1.389 (4)
N2A—C1A	1.336 (3)	C2B—C7B	1.397 (3)
N2A—C7A	1.383 (3)	C3B—C4B	1.383 (5)
N2A—H2AA	0.8600	C3B—H3BA	0.9300
N3A—C9A	1.332 (3)	C4B—C5B	1.393 (6)
N3A—C10A	1.334 (4)	C4B—H4BA	0.9300
C2A—C3A	1.389 (4)	C5B—C6B	1.387 (5)
C2A—C7A	1.397 (3)	C5B—H5BA	0.9300
C3A—C4A	1.381 (5)	C6B—C7B	1.376 (4)
C3A—H3AA	0.9300	C6B—H6BA	0.9300
C4A—C5A	1.399 (4)	C8B—C9B	1.502 (3)
C4A—H4AA	0.9300	C8B—H8BA	0.9700
C5A—C6A	1.384 (4)	C8B—H8BB	0.9700
C5A—H5AA	0.9300	C9B—C13B	1.389 (3)
C6A—C7A	1.390 (4)	C10B—C11B	1.371 (4)
C6A—H6AA	0.9300	C10B—H10B	0.9300
C8A—C9A	1.505 (4)	C11B—C12B	1.389 (3)
C8A—H8AA	0.9700	C11B—H11B	0.9300
C8A—H8AB	0.9700	C12B—C13B	1.387 (4)
C9A—C13A	1.401 (3)	C13B—C14B	1.509 (3)
C10A—C11A	1.374 (4)	C14B—H14D	0.9600
C10A—H10A	0.9300	C14B—H14E	0.9600
C11A—C12A	1.387 (4)	C14B—H14F	0.9600
C11A—H11A	0.9300	C15B—C16B	1.502 (3)
C12A—C13A	1.396 (4)	C15B—H15C	0.9700
C13A—C14A	1.498 (3)	C15B—H15D	0.9700
			
C6—C1—C2	123.92 (18)	F2AA—C16A—F1AA	109.21 (12)
C6—C1—Cl1	117.53 (15)	F2A—C16A—F1AA	85.83 (17)
C2—C1—Cl1	118.44 (15)	F3A—C16A—F1AA	130.4 (2)
O2—C2—C1	124.80 (18)	F3AA—C16A—C15A	107.4 (2)
O2—C2—C3	117.51 (17)	F2AA—C16A—C15A	111.1 (3)
C1—C2—C3	117.69 (17)	F1A—C16A—C15A	109.2 (3)
O3—C3—C4	125.85 (18)	F2A—C16A—C15A	113.2 (3)
O3—C3—C2	116.08 (17)	F3A—C16A—C15A	106.6 (3)
C4—C3—C2	118.06 (16)	F1AA—C16A—C15A	110.1 (2)
C3—C4—C5	124.00 (18)	C1B—S1B—C8B	99.87 (11)
C3—C4—Cl2	118.02 (14)	C12B—O1B—C15B	118.0 (2)
C5—C4—Cl2	117.81 (15)	C1B—N1B—C2B	109.11 (19)
O5—C5—C4	124.93 (18)	C1B—N1B—H1BA	125.4
O5—C5—C6	117.24 (16)	C2B—N1B—H1BA	125.4
C4—C5—C6	117.83 (16)	C1B—N2B—C7B	108.40 (19)
O6—C6—C1	125.59 (18)	C1B—N2B—H2BA	125.8
O6—C6—C5	116.07 (17)	C7B—N2B—H2BA	125.8
C1—C6—C5	118.34 (16)	C10B—N3B—C9B	117.1 (2)
C1A—S1A—C8A	100.32 (11)	N1B—C1B—N2B	109.6 (2)
C12A—O1A—C15A	119.0 (2)	N1B—C1B—S1B	120.19 (17)
C1A—N1A—C2A	108.78 (19)	N2B—C1B—S1B	130.18 (17)
C1A—N1A—H1AA	125.6	N1B—C2B—C3B	131.1 (3)
C2A—N1A—H1AA	125.6	N1B—C2B—C7B	106.3 (2)
C1A—N2A—C7A	108.54 (18)	C3B—C2B—C7B	122.6 (3)
C1A—N2A—H2AA	125.7	C4B—C3B—C2B	116.0 (3)
C7A—N2A—H2AA	125.7	C4B—C3B—H3BA	122.0
C9A—N3A—C10A	117.7 (2)	C2B—C3B—H3BA	122.0
N2A—C1A—N1A	109.5 (2)	C3B—C4B—C5B	121.5 (3)
N2A—C1A—S1A	129.56 (16)	C3B—C4B—H4BA	119.2
N1A—C1A—S1A	120.94 (17)	C5B—C4B—H4BA	119.2
N1A—C2A—C3A	131.9 (2)	C6B—C5B—C4B	122.0 (3)
N1A—C2A—C7A	106.5 (2)	C6B—C5B—H5BA	119.0
C3A—C2A—C7A	121.6 (2)	C4B—C5B—H5BA	119.0
C4A—C3A—C2A	116.7 (3)	C7B—C6B—C5B	117.0 (3)
C4A—C3A—H3AA	121.6	C7B—C6B—H6BA	121.5
C2A—C3A—H3AA	121.6	C5B—C6B—H6BA	121.5
C3A—C4A—C5A	121.7 (3)	C6B—C7B—N2B	132.5 (3)
C3A—C4A—H4AA	119.2	C6B—C7B—C2B	120.9 (3)
C5A—C4A—H4AA	119.2	N2B—C7B—C2B	106.6 (2)
C6A—C5A—C4A	121.8 (3)	C9B—C8B—S1B	107.15 (16)
C6A—C5A—H5AA	119.1	C9B—C8B—H8BA	110.3
C4A—C5A—H5AA	119.1	S1B—C8B—H8BA	110.3
C5A—C6A—C7A	116.5 (3)	C9B—C8B—H8BB	110.3
C5A—C6A—H6AA	121.7	S1B—C8B—H8BB	110.3
C7A—C6A—H6AA	121.7	H8BA—C8B—H8BB	108.5
N2A—C7A—C6A	131.7 (2)	N3B—C9B—C13B	124.2 (2)
N2A—C7A—C2A	106.7 (2)	N3B—C9B—C8B	114.8 (2)
C6A—C7A—C2A	121.6 (2)	C13B—C9B—C8B	121.0 (2)
C9A—C8A—S1A	106.77 (16)	N3B—C10B—C11B	123.8 (2)
C9A—C8A—H8AA	110.4	N3B—C10B—H10B	118.1
S1A—C8A—H8AA	110.4	C11B—C10B—H10B	118.1
C9A—C8A—H8AB	110.4	C10B—C11B—C12B	117.6 (2)
S1A—C8A—H8AB	110.4	C10B—C11B—H11B	121.2
H8AA—C8A—H8AB	108.6	C12B—C11B—H11B	121.2
N3A—C9A—C13A	124.3 (2)	O1B—C12B—C13B	116.0 (2)
N3A—C9A—C8A	115.2 (2)	O1B—C12B—C11B	123.1 (2)
C13A—C9A—C8A	120.5 (2)	C13B—C12B—C11B	120.8 (2)
N3A—C10A—C11A	123.9 (2)	C12B—C13B—C9B	116.3 (2)
N3A—C10A—H10A	118.1	C12B—C13B—C14B	121.9 (2)
C11A—C10A—H10A	118.1	C9B—C13B—C14B	121.8 (2)
C10A—C11A—C12A	117.4 (3)	C13B—C14B—H14D	109.5
C10A—C11A—H11A	121.3	C13B—C14B—H14E	109.5
C12A—C11A—H11A	121.3	H14D—C14B—H14E	109.5
O1A—C12A—C11A	123.7 (2)	C13B—C14B—H14F	109.5
O1A—C12A—C13A	115.0 (2)	H14D—C14B—H14F	109.5
C11A—C12A—C13A	121.2 (2)	H14E—C14B—H14F	109.5
C12A—C13A—C9A	115.5 (2)	O1B—C15B—C16B	107.8 (2)
C12A—C13A—C14A	121.1 (2)	O1B—C15B—H15C	110.1
C9A—C13A—C14A	123.4 (2)	C16B—C15B—H15C	110.1
C13A—C14A—H14A	109.5	O1B—C15B—H15D	110.1
C13A—C14A—H14B	109.5	C16B—C15B—H15D	110.1
H14A—C14A—H14B	109.5	H15C—C15B—H15D	108.5
C13A—C14A—H14C	109.5	F2BB—C16B—F3BB	109.42 (13)
H14A—C14A—H14C	109.5	F2BB—C16B—F1BB	109.35 (13)
H14B—C14A—H14C	109.5	F3BB—C16B—F1BB	109.33 (13)
O1A—C15A—C16A	106.7 (3)	F2BB—C16B—F3B	127.6 (2)
O1A—C15A—H15A	110.4	F3BB—C16B—F3B	87.8 (2)
C16A—C15A—H15A	110.4	F3BB—C16B—F1B	140.7 (2)
O1A—C15A—H15B	110.4	F1BB—C16B—F1B	86.25 (16)
C16A—C15A—H15B	110.4	F3B—C16B—F1B	109.20 (12)
H15A—C15A—H15B	108.6	F2BB—C16B—F2B	77.19 (18)
F3AA—C16A—F2AA	109.73 (12)	F1BB—C16B—F2B	123.6 (2)
F3AA—C16A—F1A	85.21 (19)	F3B—C16B—F2B	109.08 (12)
F2AA—C16A—F1A	129.6 (2)	F1B—C16B—F2B	109.06 (12)
F3AA—C16A—F2A	128.2 (2)	F2BB—C16B—C15B	116.2 (3)
F1A—C16A—F2A	109.42 (12)	F3BB—C16B—C15B	99.7 (3)
F2AA—C16A—F3A	86.66 (18)	F1BB—C16B—C15B	112.3 (3)
F1A—C16A—F3A	109.40 (13)	F3B—C16B—C15B	108.5 (2)
F2A—C16A—F3A	109.00 (12)	F1B—C16B—C15B	107.4 (2)
F3AA—C16A—F1AA	109.29 (12)	F2B—C16B—C15B	113.5 (2)
			
C6—C1—C2—O2	178.1 (2)	C11A—C12A—C13A—C14A	−179.5 (3)
Cl1—C1—C2—O2	2.0 (3)	N3A—C9A—C13A—C12A	0.7 (4)
C6—C1—C2—C3	−1.3 (3)	C8A—C9A—C13A—C12A	−178.2 (2)
Cl1—C1—C2—C3	−177.35 (14)	N3A—C9A—C13A—C14A	−179.9 (3)
O2—C2—C3—O3	3.1 (3)	C8A—C9A—C13A—C14A	1.2 (4)
C1—C2—C3—O3	−177.56 (19)	C12A—O1A—C15A—C16A	177.9 (2)
O2—C2—C3—C4	−177.9 (2)	O1A—C15A—C16A—F3AA	175.1 (3)
C1—C2—C3—C4	1.5 (3)	O1A—C15A—C16A—F2AA	−64.9 (4)
O3—C3—C4—C5	175.6 (2)	O1A—C15A—C16A—F1A	84.2 (4)
C2—C3—C4—C5	−3.4 (3)	O1A—C15A—C16A—F2A	−38.0 (4)
O3—C3—C4—Cl2	0.4 (3)	O1A—C15A—C16A—F3A	−157.8 (3)
C2—C3—C4—Cl2	−178.54 (14)	O1A—C15A—C16A—F1AA	56.2 (4)
C3—C4—C5—O5	−175.9 (2)	C2B—N1B—C1B—N2B	0.2 (3)
Cl2—C4—C5—O5	−0.8 (3)	C2B—N1B—C1B—S1B	−177.41 (16)
C3—C4—C5—C6	4.6 (3)	C7B—N2B—C1B—N1B	−0.1 (2)
Cl2—C4—C5—C6	179.79 (14)	C7B—N2B—C1B—S1B	177.27 (18)
C2—C1—C6—O6	−176.2 (2)	C8B—S1B—C1B—N1B	−165.94 (18)
Cl1—C1—C6—O6	−0.1 (3)	C8B—S1B—C1B—N2B	17.0 (2)
C2—C1—C6—C5	2.5 (3)	C1B—N1B—C2B—C3B	179.3 (3)
Cl1—C1—C6—C5	178.64 (14)	C1B—N1B—C2B—C7B	−0.3 (3)
O5—C5—C6—O6	−4.7 (3)	N1B—C2B—C3B—C4B	−180.0 (3)
C4—C5—C6—O6	174.79 (19)	C7B—C2B—C3B—C4B	−0.3 (5)
O5—C5—C6—C1	176.48 (19)	C2B—C3B—C4B—C5B	−0.3 (6)
C4—C5—C6—C1	−4.0 (3)	C3B—C4B—C5B—C6B	0.8 (7)
C7A—N2A—C1A—N1A	0.5 (2)	C4B—C5B—C6B—C7B	−0.6 (6)
C7A—N2A—C1A—S1A	−178.46 (17)	C5B—C6B—C7B—N2B	179.9 (3)
C2A—N1A—C1A—N2A	−0.1 (2)	C5B—C6B—C7B—C2B	0.0 (5)
C2A—N1A—C1A—S1A	178.96 (15)	C1B—N2B—C7B—C6B	179.9 (3)
C8A—S1A—C1A—N2A	0.0 (2)	C1B—N2B—C7B—C2B	−0.1 (3)
C8A—S1A—C1A—N1A	−178.86 (18)	N1B—C2B—C7B—C6B	−179.8 (2)
C1A—N1A—C2A—C3A	179.8 (3)	C3B—C2B—C7B—C6B	0.5 (4)
C1A—N1A—C2A—C7A	−0.3 (2)	N1B—C2B—C7B—N2B	0.3 (3)
N1A—C2A—C3A—C4A	178.8 (3)	C3B—C2B—C7B—N2B	−179.4 (3)
C7A—C2A—C3A—C4A	−1.0 (4)	C1B—S1B—C8B—C9B	178.68 (17)
C2A—C3A—C4A—C5A	−0.4 (5)	C10B—N3B—C9B—C13B	−1.3 (4)
C3A—C4A—C5A—C6A	1.4 (6)	C10B—N3B—C9B—C8B	179.5 (3)
C4A—C5A—C6A—C7A	−0.9 (5)	S1B—C8B—C9B—N3B	−2.7 (3)
C1A—N2A—C7A—C6A	178.2 (3)	S1B—C8B—C9B—C13B	178.04 (19)
C1A—N2A—C7A—C2A	−0.7 (2)	C9B—N3B—C10B—C11B	2.5 (5)
C5A—C6A—C7A—N2A	−179.2 (3)	N3B—C10B—C11B—C12B	−0.8 (5)
C5A—C6A—C7A—C2A	−0.5 (4)	C15B—O1B—C12B—C13B	163.0 (3)
N1A—C2A—C7A—N2A	0.6 (2)	C15B—O1B—C12B—C11B	−17.4 (4)
C3A—C2A—C7A—N2A	−179.5 (2)	C10B—C11B—C12B—O1B	178.1 (3)
N1A—C2A—C7A—C6A	−178.4 (2)	C10B—C11B—C12B—C13B	−2.4 (5)
C3A—C2A—C7A—C6A	1.5 (4)	O1B—C12B—C13B—C9B	−177.0 (2)
C1A—S1A—C8A—C9A	171.22 (17)	C11B—C12B—C13B—C9B	3.4 (4)
C10A—N3A—C9A—C13A	−0.6 (4)	O1B—C12B—C13B—C14B	3.7 (4)
C10A—N3A—C9A—C8A	178.4 (2)	C11B—C12B—C13B—C14B	−175.8 (3)
S1A—C8A—C9A—N3A	10.6 (3)	N3B—C9B—C13B—C12B	−1.6 (4)
S1A—C8A—C9A—C13A	−170.4 (2)	C8B—C9B—C13B—C12B	177.5 (2)
C9A—N3A—C10A—C11A	−0.2 (4)	N3B—C9B—C13B—C14B	177.6 (3)
N3A—C10A—C11A—C12A	0.8 (5)	C8B—C9B—C13B—C14B	−3.3 (4)
C15A—O1A—C12A—C11A	10.1 (5)	C12B—O1B—C15B—C16B	−156.3 (2)
C15A—O1A—C12A—C13A	−170.4 (3)	O1B—C15B—C16B—F2BB	34.4 (4)
C10A—C11A—C12A—O1A	178.9 (3)	O1B—C15B—C16B—F3BB	−82.9 (3)
C10A—C11A—C12A—C13A	−0.6 (4)	O1B—C15B—C16B—F1BB	161.4 (2)
O1A—C12A—C13A—C9A	−179.6 (2)	O1B—C15B—C16B—F3B	−173.8 (2)
C11A—C12A—C13A—C9A	−0.1 (4)	O1B—C15B—C16B—F1B	68.3 (3)
O1A—C12A—C13A—C14A	0.9 (4)	O1B—C15B—C16B—F2B	−52.4 (3)

### Hydrogen-bond geometry (Å, °)

**Table:**

*D*—H···*A*	*D*—H	H···*A*	*D*···*A*	*D*—H···*A*
N1A—H1AA···O3	0.86	1.95	2.749 (2)	155
N1A—H1AA···Cl2	0.86	2.96	3.5169 (18)	125
N2A—H2AA···O5^i^	0.86	1.91	2.737 (2)	160
N1B—H1BA···O2	0.86	1.89	2.717 (2)	160
N2B—H2BA···O6^i^	0.86	1.96	2.766 (2)	155
C8A—H8AB···O5^i^	0.97	2.50	3.195 (3)	127
C8B—H8BA···O6^i^	0.97	2.45	3.289 (3)	145
C6B—H6BA···F3AA^ii^	0.93	2.49	3.104 (5)	124

Symmetry codes: (i) *x*+1/2, −*y*+1/2, *z*−1/2; (ii) −*x*+3/2, *y*−1/2, −*z*+1/2.

### Table 2 Weak π-π hydrogen-bond intermoleular interactions (Å)

**Table:**

Cg···Cg	D···A
Cg1···Cg4^i^	3.8187 (13)
Cg2···Cg2^ii^	3.5631 (15)
Cg2···Cg5^i^	3.7434 (17)
Cg3···Cg6^i^	3.842 (2)

Symmetry codes: (i) x, y, z; (ii) 2-x, 1-y, 1-z; Cg1,Cg2,Cg3,Cg4,Cg5,Cg6 are the centroids of the N1A/C1A/N2A/C7A/C2A; N3A/C9A/C13A/C12A/C11A/C10A; C2A–C7A; N1B/C1B/N2B/C7B/C2B; N3B/C9B/C13B/C12B/C11B/C10B; C2B–C7B rings.
